# Genetic Diversity and Allelic Distribution of *Plasmodium falciparum msp1* and *msp2* Genes in a High‐Transmission Setting: Southern Ethiopia

**DOI:** 10.1155/japr/2796576

**Published:** 2026-06-19

**Authors:** Kefiyalew Jote, Yirgalem Gebrehiwot, Cheikh Cambel Dieng, Eugenia Lo, Lemu Golassa, Bayissa Chala

**Affiliations:** ^1^ Department of Applied Biology, Adama Science and Technology University, Adama, Ethiopia, astu.edu.et; ^2^ Institute of Pathobiology, Addis Ababa University, Addis Ababa, Ethiopia, aau.edu.et; ^3^ Department of Microbiology and Immunology, College of Medicine, Drexel University, Philadelphia, Pennsylvania, USA, drexel.edu

**Keywords:** Ethiopia, genetic diversity, malaria, *msp* gene, *P. falciparum*

## Abstract

**Background:**

Although both preventable and treatable, malaria remains a significant burden on health and economic stability, particularly in regions of high transmission such as Africa and parts of Asia. The parasite′s genetic diversity enhances its ability to evade the host immune system and adapt, posing challenges to effective treatment strategies.

**Objective:**

The primary objective of this study was to assess the genetic diversity of *Plasmodium falciparum* by analyzing polymorphisms in the merozoite surface protein genes among clinical isolates collected from public health facilities in Arba Minch town and Mirab Abaya, Southern Ethiopia.

**Methods:**

A facility‐based cross‐sectional study was conducted to investigate the genetic diversity of *P. falciparum*. Capillary blood samples were collected from 200 participants using both microscope slides (for phenotypic analysis) and Whatman 903 filter paper (for molecular investigation using polymerase chain reaction). Parasite genomic DNA was extracted using the G‐spin Total DNA Extraction Mini Kit. Nested PCR was performed to confirm the presence of *P. falciparum* by targeting 18S rRNA and to genotype the *msp1 and msp2* gene allelic families.

**Results:**

Of the 200 samples initially confirmed for *P. falciparum* infection, 138 (69%) were mono‐infections. Males accounted for 55.1% of the confirmed cases, whereas 44.9% were females. More than half of the participants (58%) had parasite density of ≥ 10,000 parasites/*μ*L. The mean hemoglobin level recorded was 13.4 g/dL (95% CI, 13.06–13.7). Genotyping revealed 131 *msp1 and* 164 for *msp2* alleles, with MAD20 (58.8%) and FC27 (52.4%) frequently detected allelic types, respectively. *msp2* infections were nearly evenly split between monoclonal (53.2%) and polyclonal. A significantly higher multiplicity of infection (MOI) was observed in Arba Minch town for *msp2* (MOI = 2.19) *p* < 0.001.

**Conclusion and Recommendation:**

This study revealed substantial genetic diversity in *P. falciparum* alleles, with variations linked to geographic location, age group, and residential setting. These findings underscore the complex epidemiology of malaria in Arba Minch and the surrounding areas, emphasizing the need for geographically and demographically targeted control strategies based on local transmission patterns to guide elimination efforts. Enhancing community awareness about risk factors and vulnerable populations is critical for the success of malaria prevention programs.

## 1. Background

Malaria remains a critical global health concern, significantly contributing to morbidity and mortality worldwide [1, 2]. Although malaria is both preventable and treatable, it continues to have a profound impact on public health and economic development, particularly in sub‐Saharan Africa and parts of Asia where transmission is highest. Early diagnosis and prompt treatment are essential in reducing severe outcomes. From 2015 to 2019, global malaria incidence increased, posing new challenges to the eradication efforts and reinforcing the need for sustained research into control strategies [2].

In Ethiopia, between January 1 and October 20, 2024, over 7.3 million malaria cases and 1157 deaths were recorded, reflecting a case fatality rate of 0.02% [3]. Malaria affects approximately 75% of Ethiopia′s landmass, putting nearly 69% of the population at risk [4]. Children under five are especially vulnerable, with malaria‐related deaths accounting for an estimated 20% of mortality in this age group. Challenges to delivering effective malaria care are exacerbated in conflict‐affected areas, where access to health services is limited and resources are further strained by concurrent disease outbreaks and ongoing humanitarian crises [5].

*Plasmodium falciparum*, the most virulent human malaria parasite, exhibits substantial genetic diversity across endemic regions, shaped by factors such as transmission intensity, immune selection, geographic isolation [6], genetic recombination [7] and selective pressure [8]. This diversity is driven in part by the parasite′s capacity for antigenic switching and recombination within the mosquito gut, which enhances immune evasion and contributes to the emergence of drug resistance [9, 10]. Additionally, *P. falciparum* expresses variant surface antigens on infected red blood cells, allowing continuous immune evasion, which is critical for persistent infections [11]. Key surface proteins such as *msp1* and *msp2* play critical roles in the parasite′s invasion and immune evasion strategies, making them important targets for the host′s immune response and potential candidates for vaccine development [12].

The extensive polymorphism of *msp1* and *msp2* poses a major challenge for vaccine design, as immunity to one allelic variant may not provide cross‐protection against others [13]. This genetic variability not only enhances immune escape but also complicates treatment strategies by facilitating the selection of adaptive traits. Furthermore, previous studies have shown that the effectiveness of various interventions is influenced by time and geographic location, primarily due to genetic polymorphisms [14]. Meaning that *P. falciparum′s* genetic diversity reflects its capacity to adapt to host environments, facilitating the selection of advantageous traits, such as drug resistance and antigenic variation [15].

Therefore, characterizing the genetic diversity of *P. falciparum* is essential for understanding the factors that sustain transmission and contribute to resurgence. Such studies provide insights into the parasite′s adaptive landscape and local transmission dynamics. A deeper understanding of this diversity is crucial to informing more effective, locally tailored malaria control strategies and guiding resource allocation for elimination efforts.

## 2. Materials and Methods

### 2.1. Study Area and Population

**Figure 1:**
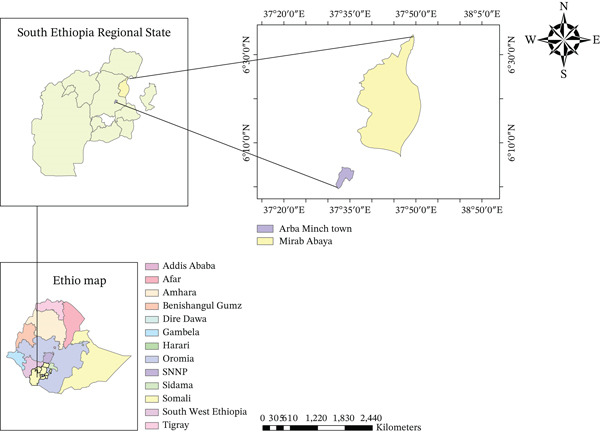
Map of the study sites as drawn using ArcGIS 9 [19].

A cross‐sectional study was conducted at three healthcare facilities in Arba Minch and Mirab Abaya, Southern Ethiopia, specifically at Woze Health Center, Dil Fana Primary Hospital both from Arbaminch town, and Birbir Health Center from Mirab Abaya. Arba Minch, the capital of Gamo Zone, located 502 km from Addis Ababa [16], has an average elevation of 1455 m [17] and experiences unstable malaria transmission, with an annual entomological inoculation rate of 17.1 infectious bites per person, primarily attributed to *P. falciparum* [18]. Mirab Abaya is located 55 km north of Arba Minch town. The locations of the study areas are shown in Figure 1.

The region′s humid and warm climate [20] favors malaria vector proliferation, making the disease endemic among local communities, which mainly consist of subsistence farmers cultivating cash crops such as mangoes and bananas. Arba Minch covers 20 districts, comprising 14 rural and 6 urban areas, and is served by 57 health centers, 6 hospitals, and 320 health posts.

We recruited a total of 200 participants with confirmed *P. falciparum* malaria from Arba Minch town and the Mirab Abaya district between May and December 2023. First, we selected three health centers, Birbir, Dil Fana, and Woze, from the facilities in both areas using a lottery method, which is a simple random sampling technique to reduce selection bias since all the health facilities in the area have high patient volumes. Second, we purposively enrolled only individuals with laboratory‐confirmed *P. falciparum* infections since our concern is determining the genetic diversity of *P. falciparum* in the study areas. To reach a sufficient sample size for robust analysis, we conveniently selected participants based on the average caseload of each facility. Consequently, we recruited 94 participants from Birbir, 56 from Woze, and 50 from Dil Fana health centers.

### 2.2. Inclusion and Exclusion Criteria

Participants were included if they consented to the study, tested positive for *P. falciparum* mono‐infection (i.e., parasite is identified exclusively as *P. falciparum,* with no mixed‐species infection), and had not used antimalarial drugs in the past 12 h. Regardless of malaria transmission intensity, people of all age groups were included except infants less than 12 months of age. Only permanent residents of the study area were considered. The residency status was determined based on information obtained during patient registration and confirmed through interviews conducted during data collection. Participants who asked to withdraw themselves from the study and samples that failed to be amplified with polymerase chain reaction (PCR) were excluded.

### 2.3. Body Temperature

Axillary temperature was obtained and recorded to the nearest 0.1°C to describe clinical features of the study participants, and fever was defined as an axillary temperature of > 37.5°C. Body temperature was not used as an inclusion or exclusion criterion; therefore, both febrile and afebrile patients were included in the study.

### 2.4. Parasitological Examination

Blood samples for microscopic examination were obtained from finger prick using an automatic blood lancet. Thin and thick blood films were prepared and stained with Giemsa stain following standard World Health Organization (WHO) guidelines [21]. Two experienced laboratory technologists examined the slides independently and they were blinded to each other′s results, demographic and clinical information of the participants to minimize observer bias. Parasite density was calculated for samples presenting with *P. falciparum* mono‐infection as previously described [22]. Briefly, it was obtained by counting the actual number of parasites in thick blood film preparation in relation to a predetermined number of WBCs and an average of 8000/*μ*L was taken as standard. Accordingly, the following formula was used:

Number of parasites/μL=number of parasites countednumber of WBCs counted∗8000 WBCs/μL

The parasite densities were classified as low, moderate, and severe as previously described somewhere else [23]. In brief, it was classified as low when parasitemia was less than 1000 parasites/*μ*L of blood; moderate when parasitemia was between 1000 and 9999 parasites/*μ*L of blood; and severe when parasitemia was greater than or equal to 10,000 parasites/*μ*L of blood.

### 2.5. Hematological Analysis and DBS Preparation

Hematological parameters were assessed by collecting 5 mL of ethylenediaminetetraacetic acid (EDTA) anticoagulated blood samples from patients. The samples were then analyzed using a Beckman Coulter counter (URIT‐3300), a reliable automated hematology analyzer known for its precision in evaluating blood cell indices. This method aligns with previously established protocols, ensuring accuracy and consistency in measuring key parameters such as red and white blood cell counts, hemoglobin levels, and platelet counts [24]. For molecular analysis, blood samples were collected on Whatman 903 filter papers from eligible participants via finger prick. The filter papers were dried, sealed in plastic bags with silica gel, and stored at ‐20°C temporarily at Woze health center, as per established protocols [25]. The coded samples were then transported to Addis Ababa University, Institute of Pathobiology for PCR‐based molecular analysis.

### 2.6. Deoxyribose Nucleic Acid (DNA) Extraction

The parasite DNA was purified using the G‐spin Total DNA Extraction Mini Kit [26] following the manufacturer′s instructions with minor modifications. The eluates were stored at −20°C for subsequent analysis.

### 2.7. Primers and PCR Conditions

The *P. falciparum* 18S ribosomal ribonucleic acid (rRNA) gene, which is highly conserved and present in multiple copies [27], was amplified using specific primers with slight modifications to previously described methods [28]. PCR products were resolved on a 1.5% agarose gel containing ethidium bromide and visualized using a Bio‐Rad UV transilluminator. Samples confirmed as *P. falciparum* mono‐infections were included in the allelic analysis. The polymorphic regions of the merozoite surface protein genes, *msp1* (Block 2) and *msp2* (Block 3), were amplified by nested PCR following a previously described protocol with minor modifications [29]. Primers and their sequences are presented in Table 1.

**Table 1:** Primers and primer sequences used to identify *msp1* and *msp2* allelic families of *P. falciparum* (2024).

Target gene/allele	Primer	PCR round	Primer sequence
*msp1*	M1‐OF	Primary	5 ^′^‐CTA GAA GCT TTA GAA GAT GCA GTA TTG‐3 ^′^
	M1‐OR		5 ^′^‐CTT AAA TAG TAT TCT AAT TCA AGT GGA‐3 ^′^
K1	K1‐F	Nest	5 ^′^‐AAT GAA GAAGAA ATT ACT CA AAA GGT GC‐3 ^′^
	K1‐R		5 ^′^‐GCT TGC ATC AGC TGG AGG GCT TGC ACC AGA‐3 ^′^
MAD20	MAD20‐F		5 ^′^‐AAA TGA AGG AAC AAG TGG AAC AGC TGT TAC‐3 ^′^
	MAD20‐R		5 ^′^‐ATC TGA AGG ATT TGT ACG TCT TGA ATT ACC‐3 ^′^
RO33	RO33‐F		5 ^′^‐TAA AGG ATG GAG CAA ATA CTC AAG TTG TTG‐3 ^′^
	RO33‐R		5 ^′^‐CAT CTG AAG GAT TTG CAG CAC CTG GAG ATC‐3 ^′^
*msp2*	M2‐OF	Primary	5 ^′^‐ATG AAG GCA ACT AAA ACA TTG TCT ATT‐3 ^′^
	M2‐OR		5 ^′^‐CTT TGT TAC CAT CGG TAC ATT CTT‐3 ^′^
3D7	3D7‐F	Nest	5 ^′^‐GCA GAA AGT AAG CCT TCT ACT GGT GCT‐3 ^′^
	3D7‐R		5 ^′^‐GAT TTG TTT CGG CAT TAT TAT‐GA‐3 ^′^
FC27	FC27‐F		5 ^′^‐GCA AAT GAA GGT TCT AAT ACT AAT AG‐3 ^′^
	FC27‐R		5 ^′^‐GCT TTG GGT CCT TCT TCA GTT GAT TC‐3 ^′^

## 3. Data Analysis

Data were entered into Epidata software Version 3.6 and subsequently exported to Statistical Software for Social Science (SPSS) Version 26 for analysis. Descriptive statistics were used to summarize sociodemographic variables including *msp1* and *msp2* gene data. The multiplicity of infection (MOI), an index of malaria transmission intensity, was calculated by dividing the total number of alleles detected for each gene by the total number of samples positive for the corresponding isolates [30]. Fisher′s exact test was employed to assess associations between categorical variables, with statistical significance set at a *p* value of < 0.05. Genetic diversity was determined using the formula:

He=nn−11−Σpi2

where He = expected heterozygosity, *n* = the number of isolates sampled, and *p*_*i*_ = allele frequency at a specific locus [30].

## 4. Results

### 4.1. Demographic Characteristics

A total of 200 samples initially diagnosed as *P. falciparum* mono‐infections by microscopy were included in the study. However, subsequent PCR analysis confirmed 138 of these cases (69%) as true *P. falciparum* mono‐infections.

The mean age of the participants was 22.4 years (± 15.5 SD), with ages ranging from 1 to 75 years. A majority of participants, 81 individuals (58.7%) reported having had malaria within the previous 12 months, and 33 of them (23.9%) experienced two or more episodes during the same period (Table 2).

**Table 2:** Sociodemographic characteristics of the study participants at Arba Minch town and Mirab Abaya, Southern Ethiopia (2024).

Characteristics of the participants	Frequency (*n*)	Percentage (%)
Locality		
Arba Minch town	78	56.5
Mirab Abaya	60	43.5
Sex		
Male	76	55.1
Female	62	44.9
Age		
Less than 5 years	15	10.9
Between 6 and 14	28	20.3
Between 15 and 34	68	49.3
Above 35	27	19.6
Had information about malaria regularly	86	62.3
Had information about drug resistant malaria	44	31.9

### 4.2. Clinical and Hematological Characteristics

The majority of the participants, 119 (86.2%) presented with an axillary temperature of ≥ 37.6°C at the time of sample collection (Table 3). Among them, only 19 (13.8%) had no headache during sample collection.

**Table 3:** Clinical and hematological characteristics of the study participants at Arba Minch town and Mirab Abaya health facilities, Southern Ethiopia, 2024.

Variables	*M* *e* *a* *n* ± *S* *D* ^a^	95% CI^b^
Body temperature	38.33 ± 0.714	38.21–38.45
Parasite density	23,217.83 ± 27,057.71	18,663.20–27772.45
White blood cell count/*μ*L	11,827.30 ± 9,561.17	10,211.90–13442.70
Red blood cell count/*μ*L	4,382,394.16 ± 874138.12	4,234,704.66–4,530,083.66
Platelet count/*μ*L	244,472.10 ± 329,571.75	188,995.25–299,948.96

^a^Standard deviation.

^b^Confidence interval.

Parasite density varied among participants: 80 individuals (58%) had parasitic density of greater than ≥ 10,000 parasites/*μ*L, whereas only 3 individuals (2.2%) had densities below 1000 parasites/*μ*L.

The mean cell volume (MCV), mean cell hemoglobin (MCH), MCH concentration, and the hemoglobin values were 90.8 ± 6.27 fL(95% CI, 89.77–91.88),31.8 ± 2.65 pg(95% CI, 31.35–32.24),35.2 ± 1.87 g/dL(95% CI, 34.8–35.5), and13.4 ± 1.91 g/dL(95% CI, 13.06–13.7), respectively.

### 4.3. Prevalence of *msp1* and *msp2* Allelic Families

Of 138 samples PCR‐confirmed *P. falciparum*, 131 were successfully amplified for *msp1 and/or msp2* genes. A total of 131 amplified fragments were detected for *msp1* and 164 alleles for *msp2*, corresponding to 74 distinct *msp1* alleles and 59 distinct *msp2* alleles.

The allelic families were classified according to their number of bands present, PCR fragments, and the number of bands observed. The overall proportion of monoclonal infection (infection by single *P. falciparum* clone) was higher in *msp1* (72.5%) compared with polyclonal infection (infection by two or more *P. falciparum* clones). For *msp2*, monoclonal infection also predominated (53.2%), though the difference was less pronounced (Table 4).

**Table 4:** Distribution of *msp1* and *msp2* allelic families among study participants of Arba Minch town and Mirab Abaya, Southern Ethiopia (2024).

Gene/allele	Positive isolates (%)	Band size	Number of observed bands
*msp1*			
K1	43 (32.5)	200–400	4
MAD20	77 (58.8)	100–450	3
RO33	11 (8.4)	150–300	2
K1/MAD20	19 (67.9)		
K1/RO33	2 (7.1)		
RO33/MAD20	7 (25)		
K1/MAD20/RO33	0 (0)		
*msp2*			
3D7	78 (47.6)	200–700	9
FC27	86 (52.4)	200–700	6
3D7 and FC27	53 (47.8)		

### 4.4. Distribution of Allelic Families Among Localities

Significant differences in the frequencies of *msp1* alleles were observed between Arba Minch town and Mirab Abaya. Specifically, the K1 and RO33 alleles, as well as the K1/MAD20 and RO33/MAD20 combinations, differed significantly between the two localities (*p* < 0.05; Table 5). In contrast, the frequency of the MAD20 allele alone did not differ significantly (*p* > 0.05).

**Table 5:** Distribution of the allele frequency for the *msp* genes of *P. falciparum* by locality, Southern Ethiopia (2024).

Gene	Allele type	Locality		*p* value
		Arba Minch town frequency (%)	Mirab Abaya frequency (%)	
*msp1*	K1	17 (39.5)	26 (60.5)	0.004
	MAD20	44 (57.1)	33 (42.9)	0.858
	RO33	10 (90.9)	1 (9.1)	0.025
	K1/RO33	1 (50.0)	1 (50)	1
	K1/MAD20	7 (36.8)	12 (63.2)	0.049
	RO33/MAD20	7 (100)	0 (0)	0.041
*msp2*	3D7	58 (67.4)	28 (32.6)	0.003
	FC27	57 (73.1)	21 (26.9)	< 0.001
	3D7/FC27	43 (81.1)	10 (18.9)	< 0.001

For *msp2*, all allele types—3D7, FC27, and the combined 3D7/FC27 genotype—showed statistically significant differences in distribution between the two sites (*p* < 0.05 for all comparisons). Each of these *msp2* alleles was more frequently detected in samples from Arba Minch town compared with those from Mirab Abaya.

### 4.5. MOI and Heterozygosity

The genetic diversity of *P. falciparum* was assessed using the Shannon heterozygosity index (He) for *msp1* and *msp2* allelic families across the two study sites. For *msp1*, Arba Minch town had an He of 0.76, whereas Mirab Abaya showed a higher diversity with an He of 0.91. In contrast, *msp2* showed comparable He values in both localities: 0.69 in Arba Minch town and 0.68 in Mirab Abaya.

The MOI was highest for *msp2* in Arba Minch town, with an average MOI of 2.19, significantly greater than that observed in Mirab Abaya (*p* <0.05). Overall, the combined MOI for *msp1* and *msp2* allelic families also showed a statistically significant difference between the two sites (*p* = 0.004) (Table 6).

**Table 6:** Multiplicity of infection and heterozygosity index of *msp1* and *msp2* by localities, Southern Ethiopia (2024).

Gene	Arba Minch town	Mirab Abaya	*p* value
*msp1*			1
MOI	1.54	1.55	
He	0.76	0.91	
*msp2*			< 0.001
MOI	2.19	1.51	
He	0.69	0.68	
Overall MOI	2.45	1.95	0.004

### 4.6. Distribution of *msp1* and *msp2* Allelic Families by Age Group of Participants

Analysis of *msp1* allelic families among different age groups revealed that monoclonal infections with MAD20 are the most prevalent followed by K1 among the age group 15–34 years, with lower proportions observed in the younger (6–14 years) and older (above 35 years) individuals. RO33 exhibited a higher prevalence among younger individuals, particularly those under 5 years and in the 6–14 years age group, while still maintaining a significant presence in the 15–34 years cohort. Polyclonal infections involving K1/MAD20 are primarily found in the younger age groups, with limited presence among older participants. Notably, the combinations K1/RO33 and RO33/MAD20 were dominant in the 15–34 years age group, although both showed lesser contributions from younger and older groups. Importantly, a polyclonal infection encompassing all three alleles (K1/MAD20/RO33) was not detected across all age groups.

**Figure 2:**
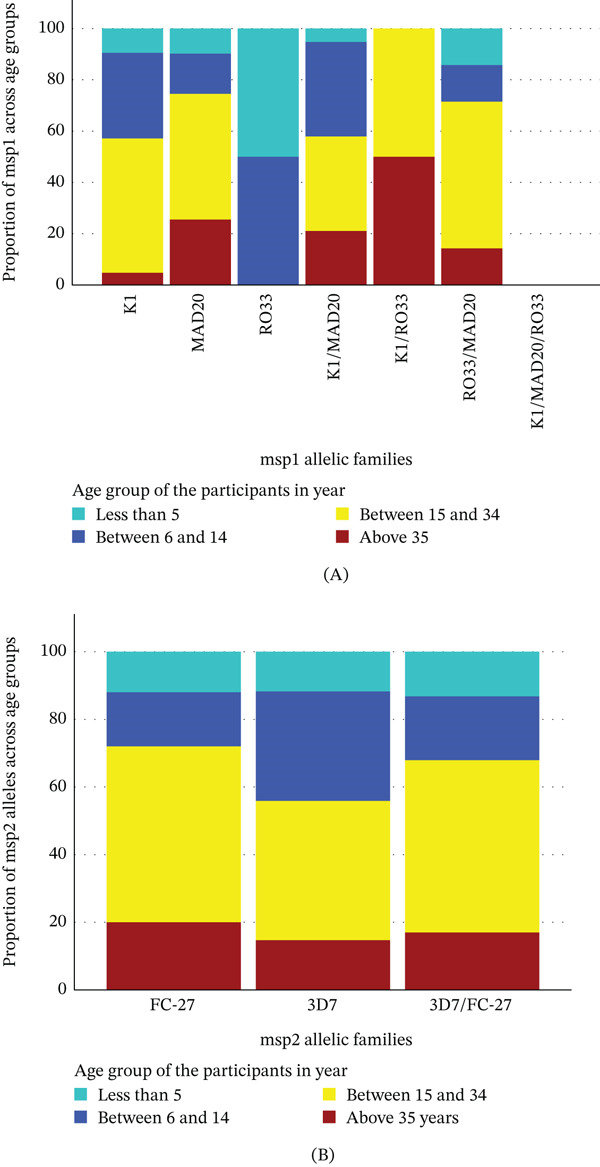
Distribution of the *msp1* and *msp2* allelic families according to age groups of the participants at Arba Minch and Mirab Abaya health facilities, Southern Ethiopia. (A) *msp1*—distribution across age groups. (B) *msp2*—distribution across age groups.

On the other hand, the distribution of *msp2* alleles among different age groups revealed that individuals aged 15–34 years harbored a significant proportion of monoclonal infections with FC27 and 3D7 alleles. Similarly, the age group 6–14 years also showed a considerable presence of these alleles, whereas participants above 35 years and those younger than 5 years old represented smaller proportions. Polyclonal infections comprising both 3D7 and FC‐27 demonstrated a higher prevalence in the 15–34 years group, with notable levels in the 6–14 years group as well. Distribution of the allelic proportions according to age groups is indicated in Figure 2.

### 4.7. Distribution of Infection Types According To Residence and Parasite Density

We investigated monoclonal and polyclonal *msp1* and *msp2* infections in relation to participants′ residence and parasite density (Table 7). For *msp1* allelic families, neither monoclonal nor polyclonal infections showed statistically significant differences across residence or parasite density levels.

**Table 7:** Distribution of *P. falciparum* allelic infection types according to residence and parasite density, South Ethiopia (2024).

Gene/infection type	Residence			*p* value	Parasite density			*p* value
	Urban no./%	Periurban no./%	Rural no./%		< 1000 no./%	1001–9999 no./%	> 10000 no./%	
*msp1*								
Monoclonal	36/48.6	18/24.3	20/27.0	0.762	1/1.4	34/45.9	39/52.7	0.156
Polyclonal	16/57.1	3/10.7	9/32.1	0.928	2/7.1	9/32.1	17/60.7	0.686
*msp2*								
Monoclonal	38/64.4	7/11.9	14/23.7	0.044	1/1.7	27/45.8	31/52.5	0.222
Polyclonal	19/35.8	12/22.6	22/41.5	0.001	1/1.9	10/18.9	42/79.2	<0.001

For *msp2* infections, monoclonal infections were significantly more common among urban participants, with 64.4% exhibiting single‐clone infections (*p* = 0.044). However, no significant association was found between monoclonal infections and parasite density (*p* = 0.222).

In contrast, polyclonal *msp2* infections showed significant variation by both residence and parasite density. Higher frequencies of polyclonal infections were observed in rural areas (*p* = 0.001) and among individuals with high parasite densities (> 10,000 parasites/*μ*L: *p* < 0.001).

## 5. Discussion

The findings of this study provide important insights into the demographic and molecular characteristics of *P. falciparum* mono‐infections among patients attending public health facilities in the Arba Minch and its surrounding area. Of the 200 samples initially diagnosed as *P. falciparum*–positive by microscopy, only 138 (69%) were confirmed as mono‐infections by PCR. This inconsistency signifies limitations in the diagnostic accuracy of microscopy when used as the sole method, particularly in contexts where mixed infections or low parasitemia may occur. In this regard, molecular tools such as PCR provide greater sensitivity and specificity complementing routine diagnosis, particularly in epidemiological and surveillance studies [31].

Previous studies have demonstrated that sole reliance on microscopy can lead to underdiagnosis of submicroscopic infections and misclassification of mixed infections, which are frequent in high‐transmission areas [32]. Likewise, this study revealed limitations in detecting and identifying malaria parasites which may have affected appropriate care for other febrile illness. This could also result in unnecessary treatment with antimalarial drugs which undoubtedly increase healthcare costs and contribute to the growing threat of drug resistance [33].

Despite the majority of the participants having access to information about malaria, only a small proportion of the study participants were informed about drug resistant malaria in general (Table 2). In such cases, people are unlikely to understand the importance of adhering to full drug regimens or may unintentionally rely on ineffective drugs which may lead to the spread of resistant strains in either case [34, 35]. On the other hand, the correlations between fever and high parasitic density observed in this study strengthen the link between parasitemia and clinical manifestations [36, 37].

In addition to the clinical observations described above, the elevated mean value of WBCs, combined with variations in RBCs and platelet counts, were observed among some participants (Table 3). These findings align with various studies where alterations in such hematological parameters were obtained in uncomplicated malaria cases [34, 38, 39]. Unlike some reports showing normal or reduced WBC counts in uncomplicated falciparum malaria [40, 41], the elevated mean WBC count obtained here suggests the role of white blood cells in fighting against malaria or concomitant bacterial infections, although this was not systematically investigated in this study. Nevertheless, this is supplemented by other studies where higher WBC counts were observed with increasing parasite density [42, 43].

Comparative studies in malaria cases showed lower RBC, hemoglobin, and hematocrit values compared with controls with severity increasing at higher parasite densities [41, 44]. In contrast, this study provided values within commonly reported reference ranges for RBC, hemoglobin, and hematocrit. This aligns with similar findings obtained in a study on hematological parameters variations among patients with uncomplicated *P. falciparum* infection [45]. Despite the presence of thrombocytopenia in most *P. falciparum* infected cases [46], this study showed a mean platelet count closer to normal although individual variation was considerable. Such hematological findings may be due to the predominance of mild disease, early presentation, or population‐specific factors underscoring the need to interpret hematological changes alongside clinical presentation and local reference data for uncomplicated malaria.

A moderate allelic diversity was observed for *msp1* (27.5%), with K1/MAD20 combination accounting for 67.9% within this polyclonal infection. On the other hand, a notable level of parasitic genetic diversity was detected for *msp2*, where the polyclonal infection accounted for 47.8% (Table 4). This reflects considerable genetic variability of the parasite and such variation in these loci is a key concern in malaria research, as it facilitates immune evasion and imposes challenges for vaccine development [47]. The high prevalence of MAD20 in *msp1*, and FC27 in *msp2* alleles observed in this study aligns with findings from other endemic regions where such alleles are commonly reported with demonstrable allele distribution patterns that may be affected by epidemiological and local immunological factors [48–50]. In contrast, findings from nearby areas like Kolla‐Shele, Ethiopia, showed the predominance of 3D7 indicating regional variability in *msp2* alleles [30].

Although monoclonal infections were slightly more frequent, particularly for *msp2* (53.2%), the concomitant presence of polyclonal infections and the observed MOI values indicate active and heterogeneous transmission. This implies that individual infections may be more frequently monoclonal in some areas, whereas the parasite maintains overall considerable allelic diversity. This underscores the need for assessing multiple metrics that include monoclonal proportion, MOI, and heterozygosity when characterizing transmission dynamics [50, 51].

A significant spatial allelic variation in *msp1* and *msp2* has been obtained between the two localities, Arba Minch town and Mirab Abaya (Table 5). This finding supports a study conducted in Metehara, Ethiopia, where MAD20 often predominates among *msp1* alleles, although K1 allele is also reported most frequently [52]. The statistically significant distribution of 3D7, FC27, and 3D7/FC27 clones between the two localities illustrates the genetic heterogeneity of *P. falciparum* in the area. This may be attributed to variations in vector ecology, localized transmission dynamics, host‐related factors, immunity and population movement, consequently influencing parasite diversity and selection pressure [53–55]. Similarly, this study also revealed locality‐based significant variation in *msp1* allelic infections. In particular, K1, RO33, and mixed allelic combinations (K1/MAD20 and RO33/MAD20) suggest the presence of complex multiclonal infections. Such allelic differences are frequently observed in moderate to high transmission settings, where repeated exposure facilitates coinfection with multiple parasite strains [56, 57].

The elevated MOI observed in Arba Minch town (MOI = 2.19 for *msp2*) shows greater genetic complexity of *P*. *falciparum* infections in this setting (Table 6). Together with the statistically significant difference in MOI between study sites (*p* < 0.05), the findings of this study suggest differences in transmission dynamics between the areas. Various studies showed that higher MOI has been reported notably from regions with more intense transmission, where people are subjected to multiple parasite clones [58–60]. Likewise, the higher He for *msp1* detected in Mirab Abaya, combined with comparable He values for *msp2* in both sites, demonstrates variations in parasite population structure throughout the study localities. Such variations may be influenced by various heterogeneous factors such as vector control strategies, healthcare access, environmental conditions, and levels of acquired immunity [61–63]. The observed MOI and He values are generally consistent with ongoing malaria transmission as indicated in several similar studies [49, 51, 57, 64]. This calls for continued molecular surveillance to better understand parasite population dynamics.

Furthermore, analysis of the *msp1* and *msp2* allelic families in this study showed disproportionate distribution of the alleles among different age groups (Figure 2), with younger and middle‐aged participants exhibiting a higher prevalence of specific variants, which has been indicated to experience more reinfections [65]. In malaria‐endemic regions, children and young adults usually develop only partial immunity, making them more susceptible to recurrent infections by genetically distinct parasite clones [66, 67]. The higher prevalence of RO33 in younger individuals might suggest age‐specific immune responses, as prior studies have shown that age influences allele‐specific immunity, potentially affecting clinical outcomes [68, 69]. However, the cross‐sectional nature of this study edges the capability to differentiate between clonal persistence and reinfections. Given that younger populations bear a disproportionate burden of malaria, targeted interventions, such as preventive therapies for children and adolescents, could reduce infection rates in these age groups [70].

Concerning distribution of infection types according to residence and parasite density, *msp1* clonal infections do not vary significantly by residence and parasite density, whereas *msp2* infections showed significant differences across these factors (Table 7). This finding aligns with a study where *msp2* diversity and MOI tend to be higher in high‐transmission settings [51, 71, 72] and correlate positively with parasite density [72], demonstrating more complex infections under intense transmission. The findings generally indicate considerable genetic diversity of *P. falciparum* which needs continued molecular surveillance to better inform targeted malaria control strategies.

## 6. Conclusion and Recommendation

This study revealed substantial genetic diversity in *P. falciparum* alleles, with significant variation across localities, age groups, and residential settings, underscoring the complex and localized epidemiology of malaria in Arba Minch and Mirab Abaya. These findings highlight the importance of designing targeted malaria control strategies that account for regional genetic profiles and transmission dynamics.

Raising community awareness about risk factors, particularly those related to living environments and vulnerable populations, is critical to reducing infection rates. Future research should focus on elucidating the mechanisms underlying the emergence of drug resistance and further characterizing transmission patterns in the region. Enhanced genetic surveillance, combined with locally tailored interventions, will be essential to sustaining progress toward malaria control and eventual elimination.

**Nomenclature**
ASTU: Adama Science and Technology University
DNA: deoxyribose nucleic acid
EDTA: ethylenediaminetetraacetic acid
IRB: Institutionl Review Board
MCH: mean corpouscular hemoglobin
MCHC: mean corpouscular hemoglobin concentration
MCV: mean corpouscular volume
MOI: multiplicity of infection
PCR: polymerase chain reaction
RBC: red blood cell
rRNA: ribosomal ribonucleic acid
SPSS: Statistical Package for Social Science
WBC: white blood cell
WHO: World Health Organization

## Author Contributions

K.J. conceived the idea, prepared the proposal, and conducted data collection, analysis, and write‐up of the results. B.C. and L.G. supervised the proposal, data collection, analysis, and write‐up of the results. Y.G. analyzed and interpreted the data. E.L. and C.C.D. contributed a lot in writing the manuscript.

## Funding

No funding was received for this manuscript.

## Disclosure

All authors read and approved the final manuscript.

## Ethics Statement

Ethical approval for the study was obtained from the University Ethical Review Board (UERB) with the ethics approval number of RECSoANS/BIO/10/2022 from Adama Science and Technology University (ASTU). Additionally, a letter of authorization to commence the study was secured from the Arba Minch Zonal Health Bureau. We recruited patients who attended outpatient departments to seek medical attention for acute febrile illness. All patients with febrile illness who underwent blood examination as part of their own routine clinical management were conveniently selected. Thus, the study did not pose any additional risk. We obtained verbal informed consent from the participants prior to blood collection. Verbal informed consent was preferred to avoid disrupting the clinical workflow at busy outpatient departments and prevent potential coercion that could arise from presenting a written consent to patients in need of medical attention. A trained data collector explained the purpose of the study, procedures, and the voluntary nature of the participation safeguarding confidentiality. A checklist prepared for this purpose was used to verify verbal agreement of the participant. The consent was obtained in the local language to avoid a communication barrier with the presence of a designated witness, laboratory professionals working at the study facilities. For children participants, informed assent was obtained from their parents or legal guardians to ensure adherence to ethical standards. All methods were performed in accordance with the relevant guidelines and regulations.

## Consent

The authors have nothing to report.

## Conflicts of Interest

The authors declare no conflicts of interest.

## Data Availability

The data that support the findings of this study are available on request from the corresponding author. The data are not publicly available due to privacy or ethical restrictions.
